# Cell-Specific Gene Deletion Reveals the Antithrombotic Function of COX1 and Explains the Vascular COX1/Prostacyclin Paradox

**DOI:** 10.1161/CIRCRESAHA.119.314927

**Published:** 2019-09-12

**Authors:** Jane A. Mitchell, Fisnik Shala, Youssef Elghazouli, Timothy D. Warner, Carles Gaston-Massuet, Marilena Crescente, Paul C. Armstrong, Harvey R. Herschman, Nicholas S. Kirkby

**Affiliations:** 1 From the National Heart and Lung Institute, Imperial College London, United Kingdom (J.A.M., F.S., Y.E., N.S.K.); 2 Blizard Institute (T.D.W., M.C., P.C.A.), Queen Mary University of London, United Kingdom; 3 Centre for Endocrinology, William Harvey Research Institute (C.G.-M.), Queen Mary University of London, United Kingdom; 4 Department of Molecular and Medical Pharmacology, David Geffen School of Medicine, University of California Los Angeles (H.R.H.).

**Keywords:** eicosanoids, endothelium, phenotype, platelet aggregation, thrombosis

## Abstract

Endothelial cells (ECs) and platelets, which respectively produce antithrombotic prostacyclin and prothrombotic thromboxane A_2_, both express COX1 (cyclooxygenase1). Consequently, there has been no way to delineate any antithrombotic role for COX1-derived prostacyclin from the prothrombotic effects of platelet COX1. By contrast, an antithrombotic role for COX2, which is absent in platelets, is straightforward to demonstrate. This has resulted in an incomplete understanding of the relative importance of COX1 versus COX2 in prostacyclin production and antithrombotic protection in vivo.

**Objective:**

We sought to identify the role, if any, of COX1-derived prostacyclin in antithrombotic protection in vivo and compare this to the established protective role of COX2.

**Methods and Results:**

We developed vascular-specific COX1 knockout mice and studied them alongside endothelial-specific COX2 knockout mice. COX1 immunoreactivity and prostacyclin production were primarily associated with the endothelial layer of aortae; freshly isolated aortic ECs released >10-fold more prostacyclin than smooth muscle cells. Moreover, aortic prostacyclin production, the ability of aortic rings to inhibit platelet aggregation and plasma prostacyclin levels were reduced when COX1 was knocked out in ECs but not in smooth muscle cells. When thrombosis was measured in vivo after FeCl_3_ carotid artery injury, endothelial COX1 deletion accelerated thrombosis to a similar extent as prostacyclin receptor blockade. However, this effect was lost when COX1 was deleted from both ECs and platelets. Deletion of COX2 from ECs also resulted in a prothrombotic phenotype that was independent of local vascular prostacyclin production.

**Conclusions:**

These data demonstrate for the first time that, in healthy animals, endothelial COX1 provides an essential antithrombotic tone, which is masked when COX1 activity is lost in both ECs and platelets. These results help us define a new 2-component paradigm wherein thrombotic tone is regulated by both COX1 and COX2 through complementary but mechanistically distinct pathways.

## Abstract 2

Supplemental Digital Content is available in the text.

**Meet the First Author, see p 784**

Prostacyclin is one of the body's fundamental cardioprotective hormones and has a powerful antithrombotic role. Loss of the prostacyclin receptor (I-prostanoid receptor [IP]) in mice increases thrombosis^1^ and loss-of-function IP mutations in man are associated with atherothrombotic risk.^2^ Prostacyclin formation requires activation of COX (cyclooxygenase) enzymes, for which 2 isoforms exist.^3^ COX1 is constitutively expressed in most tissues, including the vasculature, while significant levels of COX2 are constitutively expressed only in discreet regions including the kidney.^4^

Within large arteries, both endothelial cells (ECs) and smooth muscle cells (SMCs) can produce prostacyclin, although the endothelium is assumed to be the dominant site.^3,5^ COX1 drives prostacyclin release in freshly isolated arteries and circulating levels of prostacyclin's spontaneous breakdown product (6-ketoPGF_1α_).^4,6,7^ A role for COX2 in systemic prostacyclin production has been inferred from measurements of the urinary prostacyclin metabolite, PGI-M,^8^ and data from cultured cells/tissues.^9^ However, these studies must be interpreted with caution since PGI-M does not necessarily reflect systemic vascular production^10^ and COX2 is rapidly induced in vitro.^6^

Despite its unclear contribution to systemic vascular prostacyclin production, an antithrombotic influence of constitutive COX2 is readily observed in COX2-deficient mice^8,11^ and in patients taking COX2 inhibitors.^3,12^ By contrast, no physiological antithrombotic role for COX1 in vascular cells has been shown in integrated in vivo models to date. Where this question has been studied, global COX1 inhibition reduces thrombosis.^13,14^ These observations leave a paradoxical situation wherein we know vascular COX1 generates the bulk of arterial prostacyclin but it has no proven function in antithrombotic protection. COX1 is also expressed in platelets where it is coupled to the powerful platelet activator, thromboxane A_2_. As such, the vascular COX1/prostacyclin paradox has 2 possible answers, either (1) the importance of vascular COX1-derived prostacyclin has been overstated and it does not appreciably limit thrombosis or (2) when COX1 is inhibited globally, the phenotype is so dominated by loss of platelet COX1 that an important antithrombotic role for vascular COX1-derived prostacyclin is masked.

Using previous experimental models, it has not been possible to separate vascular and platelet COX1 activity. To address this, here we have generated the first available floxed *Ptgs1* mouse line and used this as the basis for conditional knockout strains wherein COX1 is specifically deleted from (1) SMCs, (2) ECs or (3) both ECs and platelets. Using these animals, along with established endothelial COX2 knockout models, we have performed studies to identify and compare the role of vascular COX isoforms in prostacyclin generation and thrombosis. These studies demonstrate for the first time a powerful antithrombotic role for vascular COX1 that acts in concert with the cardioprotective functions of COX2.

## Methods

Detailed methods are described in the Online Data Supplement. The data that support the findings of this study are available from the corresponding author on reasonable request.

Briefly, cell-specific COX1 and COX2 knockout mice were generated using a *Cre-loxP* approach. Animal work was performed in accordance with relevant legislation and ethical review. COX expression was analyzed using immunohistochemistry and confocal microscopy. Vascular cells were isolated using fluorescence-activated cell sorting. Prostanoids were measured by ELISA. Thrombosis in vivo was measured using a FeCl_3_ carotid artery injury model. Data are presented as mean±SEM for n biological replicates.

## Results

### Relative Role of ECs Versus SMCs in Prostacyclin Generation

**Figure 1.**
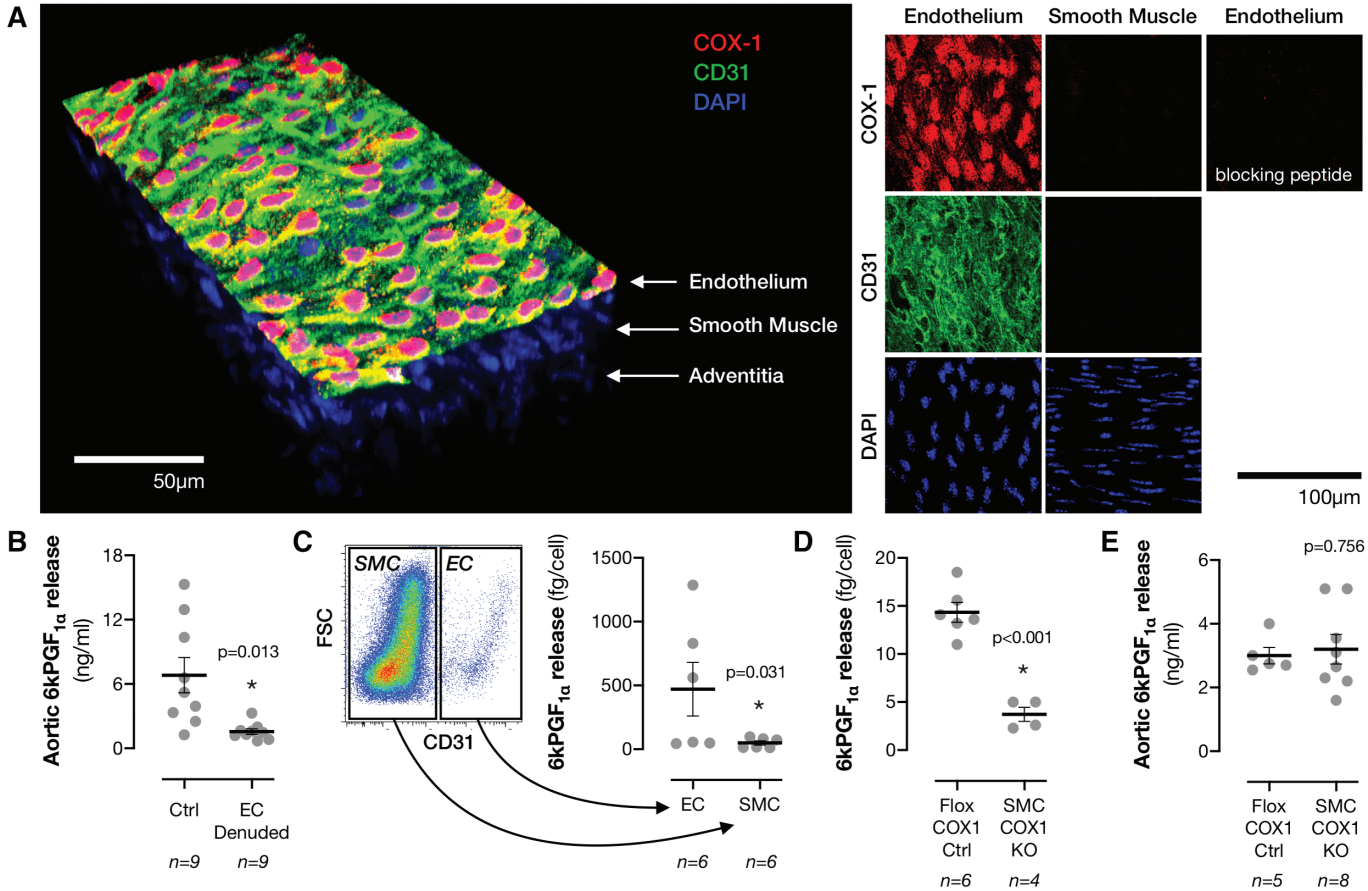
**COX1 expression and activity in vascular endothelial and smooth muscle cells.** **A**, COX1 immunoreactivity was measured through aortic rings by confocal Z-stacking microscopy and staining specificity confirmed using a blocking peptide. **B**, Prostacyclin production was measured after A23187 (30 μmol/L) stimulation in adjacent aortic rings taken from individual wild-type mice with/without mechanical endothelial removal. **C**, Endothelial and smooth muscle cells were isolated in a paired fashion from single aortas of wild-type mice using fluorescence-activated cell sorting and arachidonic acid (30 μmol/L)-induced prostacyclin release measured by immunoassay. Prostacyclin release was also measured from (**D**) aortic smooth muscle cells from Flox COX1 Ctrl and SMC COX1 KO mice and (**E**) intact aortic rings from Flox COX1 Ctrl and SMC COX1 KO mice. *P<0.05 by (**B**) paired *t* test, (**C**) paired Mann-Whitney *U* test or (**D** and **E**) unpaired *t* test.

To establish the role of vascular COX1 in antithrombotic protection, we first identified the relative contributions of COX1 in ECs and SMCs to arterial prostacyclin production. We used confocal microscopy Z-stacking to create full thickness 3-dimensional representations of the mouse aortic wall. Intense COX1 but not COX2 staining was apparent in the endothelium, consistent with our previous findings^6^; with little expression of either isoform in smooth muscle layers (Figure 1A; Online Figures I and II). This staining was blocked by an excess of the immunising peptide confirming its specificity (Figure 1A; Online Figure I). In agreement, when aortae were mechanically denuded of ECs, their ability to synthesise prostacyclin was reduced by ≈80% (Figure 1B). This observation has been made many times before, originally with the discovery of prostacyclin in 1976^15^ and, while compelling, its interpretation is based on complete, consistent removal of the endothelial layer without damage to the underlying smooth muscle. To validate this interpretation, we isolated fresh aortic ECs and SMCs using fluorescence-activated cell sorting and determined that isolated aortic ECs can generate >10-fold more prostacyclin than SMCs when activated (Figure 1C). However, it must also be considered that (1) SMCs can produce prostacyclin, albeit in small amounts, (2) that SMCs may outnumber ECs in large arteries, and (3) that the phenotype of SMCs varies between vascular beds. Therefore, the contribution to prostacyclin production in vivo may not be captured in isolated vessel/cell assays. To clarify this, we generated smooth muscle-specific COX1 knockout mice (SMC COX1 KO) by crossing floxed *Ptgs1* mice and mice carrying an *Sm22a-Cre* driver. Aortic vascular SMCs isolated from SMC KO mice (*Ptgs1*^*flox/flox*^*;Sm22a-Cre*) lost their prostacyclin synthetic capacity (Figure 1D) and *Ptgs1* mRNA expression (reduced 72±23% versus Flox COX1 Ctrl; n=2–3). In contrast, aortic ECs isolated from these mice showed no defect in prostacyclin production (Flox COX1 Ctrl: 49.8±3.3 fg/cell; SMC COX1 KO: 45.2±11.8 fg/cell; n=2–3), confirming the validity of the model. When prostacyclin generation was measured from intact aortic rings, SMC COX1 KO had no effect on prostacyclin induced by either chemical activation with Ca^2+^ ionophore A23187 (Figure 1E) or activation with mechanical agitation to mimic physical forces in the body (Flox COX1 Ctrl: 7.4±2.7 ng/mL; SMC COX1 KO: 6.7±1.2 ng/mL; *P*=0.780 by unpaired *t* test, n=5–8). Similarly, plasma levels of the stable prostacyclin breakdown product, 6-keto-PGF_1α_, an indicator of total vascular prostacyclin production in vivo, were not affected by SMC COX1 KO (Flox COX1 Ctrl: 134±8 pg/mL; SMC COX1 KO: 135±20 pg/mL; *P*=0.473 by Mann-Whitney *U* test, n=8–12). Together, these observations demonstrate that smooth muscle COX1 makes only a very minor contribution to the total vascular prostacyclin production in healthy animals. Consequently, a significant role for vascular smooth muscle COX1 in physiological prostacyclin-mediated antithrombosis can be excluded (although may warrant further investigation under conditions of vascular disease).

### Generation and Validation of Endothelial COX Knockout Mouse Models

**Figure 2.**
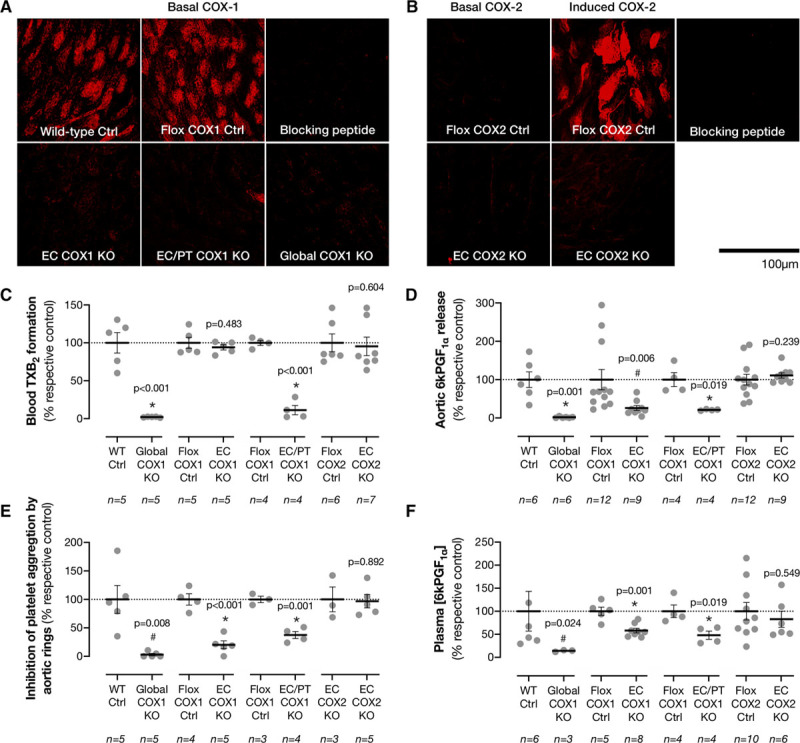
**Contribution of endothelial COX1 and COX2 to vascular and circulating prostacyclin levels.** **A**, Basal COX1 immunoreactivity was examined in the endothelial cell layer of intact aortae by confocal microscopy. **B**, COX2 immunoreactivity was examined in basal conditions and after vessels were cultured to induce expression (24 h; +LPS; 1 μg/mL). Staining specificities were confirmed using specific blocking peptides. **C**, Thromboxane production was measured by immunoassay in A23187 (30 μmol/L)-stimulated whole blood. **D**, Aortic prostacyclin production was measured after A23187-stimulation by immunoassay. **E**, The ability of aortic rings to inhibit aggregation of aspirin-treated human platelets was measured by light transmission aggregometry. **F**, Circulating prostacyclin levels were measured as plasma 6-keto-PGF_1α_ by immunoassay. In each case, data from Global COX1 KO, endothelial cell (EC) COX1 KO, EC/platelet (PT) COX1 KO, and EC COX2 KO are presented normalized to their respective control strains (100%). Absolute values for each strain and its respective control are given in Online Table I. *P<0.05 by unpaired *t* test, #*P*<0.05 by Mann-Whitney *U* test vs respective control strain.

We therefore focused on the endothelium and generated 3 additional conditional knockout models. The first, a selective endothelial COX1 knockout mouse (EC COX1 KO), generated using an inducible *VE-cadherin-Cre*^*ERT2*^ driver, was used to determine if vascular EC COX1 contributes to prostacyclin generation and antithrombotic protection. The second, a combined endothelial/platelet COX1 knockout mouse (EC/platelet COX1 KO), was made to address whether an antithrombotic role of endothelial COX1 is masked when COX1 is also removed from platelets. To generate this mouse, we took advantage that *Tie2/Tek* is expressed in both endothelium and megakaryocytes; as a result, *Cre* expressed under the *Tie2/Tek* promoter drives deletion of gene products from platelets as well as ECs.^16^ The third, as a point of comparison, was a selective endothelial COX2 knockout mouse (EC COX2 KO) generated using the same strategy as previous groups,^9^ by crossing floxed *Ptgs2* mice with a *Tie2/Tek-Cre* driver. Compared with respective controls, EC COX1 KO (*Ptgs1*^*flox/flox*^*;VE-cadherin-Cre*^*ERT2*^), EC/platelet COX1 KO (*Ptgs1*^*flox/flox*^*;Tie2-Cre*), and Global COX1 KO (*Ptgs1*^−/−^) but not EC COX2 KO (*Ptgs2*^*flox/flox*^*;Tie2-Cre*) mice demonstrated loss of aortic endothelial COX1 immunoreactivity when evaluated by confocal microscopy (Figure 2A; Online Figures I and III). Because basal COX2 expression is not detectable in aortic endothelium by this approach, to validate EC COX2 KO mice, we induced COX2 by overnight culture. This treatment resulted in robust COX2 expression which was absent in aortae from EC COX2 KO mice but fully retained in each COX1 KO strain (Figure 2B; Online Figure I). EC/platelet COX1 KO and Global COX1 KO mice also exhibited loss of platelet COX1 activity, whereas, as expected, the capacity of blood platelets to synthesise thromboxane was retained in EC COX1 KO and EC COX2 KO mice (Figure 2C; Online Table I).

Using these models, we determined the impact of endothelial COX1 and COX2 deficiency on prostacyclin generation. We observed ≈75% reduction in prostacyclin (6-keto-PGF_1α_) release from Ca^2+^ ionophore-activated aortae isolated from EC COX1 KO and EC/platelet COX1 KO mice and a complete loss in aortae isolated from Global COX1 KO mice (Figure 2D; Online Table I). In contrast, EC COX2 deletion had no effect on aortic prostacyclin release (Figure 2D; Online Table I). These results are consistent with previous findings that aortic prostacyclin synthesis requires COX1^4, 6, 7^ and is principally associated with ECs.^5^ Similar results were obtained in each model when vessels were activated by physical force (Online Table I). However, loss of prostacyclin inferred from reduced immunoreactive 6-keto-PGF_1α_ may not correlate with the antithrombotic function. To explore this question further and to validate our models, we set up a bioassay system where we could use the ability of aortic rings to inhibit platelet aggregation as a direct functional readout of their capacity to synthesise bioactive prostacyclin; analogous to the methodology used by Vane et al to first identify the antiplatelet effects of prostacyclin.^15^ Incubation of aortic rings from control animals with human platelet-rich plasma produced ≈40% inhibition of aggregation (Figure 2E; Online Table I). This inhibitory effect was associated with aortic COX1 products; inhibition of platelet aggregation was absent when Global COX1 KO aortae were used. Blocking platelet COX1 had no effect on the inhibitory activity of aortic rings in this assay (Online Figure IV) suggesting that transcellular metabolism of platelet-derived intermediates^17^ cannot support prostacyclin production in these conditions. Aortae from EC COX1 KO and EC/platelet COX1 KO mice demonstrated a blunted ability to inhibit platelet aggregation whereas EC COX2 KO retained their platelet inhibitory effect (Figure 2E; Online Table I). Across each strain, the level of platelet inhibition closely correlated (r^2^=0.96) with level of immunoreactive 6-keto-PGF_1α_, suggesting that this effect reflects a loss of the capacity to synthesise bioactive prostacyclin (Online Figure V). Next, because local production by the aorta may not fully represent prostacyclin production in all vascular beds, we extended our observations to measure plasma levels of 6-keto-PGF_1α_ in these mouse models. EC COX1 KO and EC/platelet COX1 KO but not EC COX2 KO animals exhibited a reduction in circulating (6-keto-PGF_1α_; Figure 2F; Online Table I). As expected, the remaining 6-keto-PGF_1α_ was lost in Global COX1 KO mice, indicating that COX1 in other cell types makes a contribution to total synthesis.

### Effect of Endothelial COX Deletion on Thrombus Formation In Vivo

**Figure 3.**
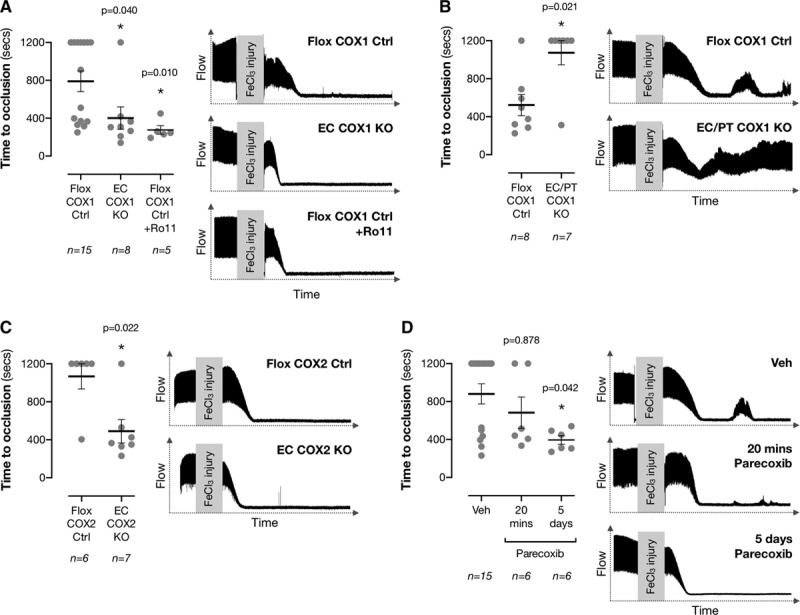
**Contribution of endothelial COX1 and COX2 to control of thrombotic tone.** Thrombosis was measured using a FeCl_3_ carotid artery injury model in (**A**) Flox COX1 Ctrl, endothelial cell (EC) COX1 KO, and Flox COX-1 Ctrl mice treated with the selective I-prostanoid receptor antagonist Ro1138452 (Ro11; 10 mg/kg; iv, 20 min), (**B**) in Flox COX1 Ctrl and EC/platelet (PT) COX1 KO mice, (**C**) in Flox COX2 Ctrl and EC COX2 KO mice, and (**D**) in wild-type mice treated with the selective COX2 inhibitor parecoxib either acutely (5 mg/kg; intravenous, 20 min) or chronically (25 mg/kg/day; oral, 5 days). Representative traces inset (scale: flow, 0–2 mL/min; time, 0–1200 s). *P<0.05 by (**A** and **D**) Kruskal-Wallis ANOVA with Dunn posttest or (**B** and **C**) Mann-Whitney *U*-test.

Having shown that EC COX1 KO mice exhibit loss of arterial prostacyclin production and that aortae from these mice lose antiplatelet activity in vitro, we used our mouse models to delineate the role of endothelial and platelet COX1 in thrombosis in vivo. Thrombosis was measured using a carotid artery FeCl_3_-injury model that has been used previously to establish the prothrombotic effects of both lP receptor deletion^1^ and global COX2 inhibition/deletion.^11,18^ EC COX1 KO mice exhibited accelerated thrombotic occlusion of the carotid artery following FeCl_3_ injury as compared with floxed littermate controls (Figure 3A), revealing the antithrombotic activity of vascular COX1. This acceleration of thrombosis was similar to that observed when floxed control mice were treated acutely with the IP receptor antagonist, Ro1138452 (Figure 3B). However, when COX1 was deleted in both ECs and platelets in EC/platelet COX1 KO mice, thrombotic occlusion time was increased (Figure 3C), despite >80% loss of prostacyclin production from carotid artery rings at the site of injury (Flox COX1 Ctrl: 10.2±3.3 ng/mL; EC/platelet COX1 KO: 2.0±1.3 ng/mL; *P*=0.010 by Mann-Whitney *U* test; n=5–7). We compared the effect of these COX1 deletions to the effect of endothelial COX2 deletion which, as previously reported,^9,18^ accelerated thrombosis after FeCl_3_ injury (Figure 3D). However, this prothrombotic effect was not associated with any loss of local prostacyclin production in the carotid artery (Flox COX2 Ctrl: 10.6±3.4 ng/mL; EC COX2 KO: 10.8±2.2 ng/mL; *P*=0.414 by Mann-Whitney *U* test; n=6–8). Acute treatment (20 minutes; ≈10× the half-life of prostacyclin) of mice with a selective COX2 inhibitor, parecoxib, to remove short-lived COX2-derived prostaglandins had no effect on thrombosis (Figure 3E). Only when parecoxib was administered chronically (5 days), at a dose producing equivalent COX2 inhibition (Online Figure VI) was the established prothrombotic effect of COX2 inhibition seen (Figure 3F).

## Discussion

By the 1970s, it was firmly established that the endothelium provides an antithrombotic surface. This observation was rapidly followed by the discovery of 2 COX products with striking opposing effects on thrombosis: prothrombotic thromboxane A_2_ and antithrombotic prostacyclin. Since this time, the prostacyclin-thromboxane balance has become a cardinal feature of cardiovascular biology and underpins the pharmacology of some of the world's most widely used drugs including aspirin and other nonsteroidal anti-inflammatory drugs.

Beyond this simple idea, much complexity has emerged. The most controversial questions surround what role, if any, vascular COX1 plays in antithrombotic protection and how this complements the well-known antithrombotic actions of COX2 seen in animal models^8,11^ and patients taking COX2 inhibitors.^3,12^ COX1 is required for prostacyclin formation by most arteries.^4,6,7^ However, to date, there have been no tools to dissociate vascular from platelet COX1 and, as such, no way to determine whether COX1-derived prostacyclin is physiologically important in thrombotic protection. In the current study, we have confirmed earlier observations using isolated cells,^5^ to show the endothelial layer is the principal site for prostacyclin generation and antiplatelet activity of intact blood vessels in vitro. Using novel conditional knockout mice, we found that endothelial-specific COX1 deletion prevented local vascular and circulating prostacyclin production and produced a prothrombotic phenotype similar to that seen when prostacyclin receptors are blocked. However, when thrombosis was studied in dual endothelial/platelet COX1 knockout mice the net effect was antithrombotic. These data demonstrate, for the first time, that endothelial COX1 provides an essential antithrombotic tone, but when COX1 activity is lost in both ECs and platelets the resulting phenotype is driven by loss of prothrombotic prostanoids from platelets. In parallel experiments using endothelial-specific COX2 knockout mice, we observed a prothrombotic phenotype, in agreement with previous reports that deletion/inhibition of COX2 in mice, either globally,^8,11^ or selectively from vascular cells,^9,18^ or renal interstitial cells increases thrombosis.^18^ However, this was not associated with any change in aortic, carotid, or circulating prostacyclin levels, consistent with observations that constitutive COX2 expression is restricted to specific tissues and vascular beds and absent in most systemic blood vessels.^4^ Further, acute administration of a selective COX2 inhibitor to remove COX2-derived prostacyclin and other short-lived prostaglandins had no effect on thrombosis. Instead, and in agreement with published protocols,^8,11^ the prothrombotic effect of parecoxib took several days to develop. Therefore, the antithrombotic effect of endothelial COX2 cannot be explained by an acute prostacyclin-mediated event such as platelet inhibition. It is important to note that in vascular disease there may be changes in the expression of COX isoforms that alter their relative roles in antithrombotic protection; however, even in severe atherosclerosis, COX1 remains the dominant driver of prostacyclin production by systemic arteries^19,20^ with a contribution from COX2 only appearing under conditions of endotoxemia.^21^

With the role for COX1 in systemic vascular prostacyclin production and antithrombotic protection established in the current study and directly compared with that of endothelial COX2, we now propose a 2-component paradigm for COX-mediated antithrombotic protection. We suggest that (1) COX1 expressed throughout the systemic vasculature generates a direct prostacyclin-mediated inhibition of platelet reactivity and (2) COX2 expressed in vascular and nonvascular cells in discrete tissue locations produces an equally powerful but indirect regulation of systemic thrombotic tone. These concepts and the availability of animal models to dissect tissue-specific roles of COX1 and COX2 should provide the impetus for renewed research into one of the body's most basic cardioprotective pathways and emphasise that, in future, we must consider the role of both COX isoforms equally if we are fully understand the cardiovascular biology of COX enzymes and the consequences of their inhibition.

## Acknowledgments

We thank Ms Jane Srivastava for assistance with cell isolation and acknowledge the Imperial College Facility for Imaging by Light Microscopy (FILM).

## Sources of Funding

This work was supported by the British Heart Foundation (FS/16/1/31699 to N.S. Kirkby; RE/13/4/30184 to J.A. Mitchell and N.S. Kirkby; RG/18/4/33541 to J.A. Mitchell and N.S. Kirkby; PG/17/40/33028 to T.D. Warner; PG/15/79/31777 to T.D. Warner), Action Medical Research (GN2272 to C. Gaston-Massuet), National Institutes of Health/National Cancer Institute (R01-CA084572, R01-CA123055, P50-CA086306; all to H.R. Herschman) and the Phelps Family Foundation and the Crump Family Foundation (to H.R. Herschman).

## Disclosures

None.

## Supplementary Material

## Novelty and Significance

### What Is Known?

- COX (cyclooxygenase) enzymes exist as 2 isoforms and generate prostanoid mediators including antithrombotic prostacyclin in blood vessels and prothrombotic thromboxane in platelets.
- COX2 is sparsely expressed in most blood vessels but has a clear antithrombotic role, evidenced by the cardiovascular side effects of drugs that inhibit COX2.
- By contrast, COX1 is the principal driver of prostacyclin in most blood vessels but paradoxically no antithrombotic role for COX1 has been demonstrated to date.

## References

[1] Murata T, Ushikubi F, Matsuoka T, Hirata M, Yamasaki A, Sugimoto Y, Ichikawa A, Aze Y, Tanaka T, Yoshida N (1997). Altered pain perception and inflammatory response in mice lacking prostacyclin receptor.. Nature.

[2] Arehart E, Stitham J, Asselbergs FW, Douville K, MacKenzie T, Fetalvero KM, Gleim S, Kasza Z, Rao Y, Martel L (2008). Acceleration of cardiovascular disease by a dysfunctional prostacyclin receptor mutation: potential implications for cyclooxygenase-2 inhibition.. Circ Res.

[3] Mitchell JA, Kirkby NS (2019). Eicosanoids, prostacyclin and cyclooxygenase in the cardiovascular system.. Br J Pharmacol.

[4] Kirkby NS, Zaiss AK, Urquhart P, Jiao J, Austin PJ, Al-Yamani M, Lundberg MH, MacKenzie LS, Warner TD, Nicolaou A (2013). LC-MS/MS confirms that COX-1 drives vascular prostacyclin whilst gene expression pattern reveals non-vascular sites of COX-2 expression.. PLoS One.

[5] Moncada S, Herman AG, Higgs EA, Vane JR (1977). Differential formation of prostacyclin (PGX or PGI2) by layers of the arterial wall. An explanation for the anti-thrombotic properties of vascular endothelium.. Thromb Res.

[6] Kirkby NS, Lundberg MH, Harrington LS, Leadbeater PD, Milne GL, Potter CM, Al-Yamani M, Adeyemi O, Warner TD, Mitchell JA (2012). Cyclooxygenase-1, not cyclooxygenase-2, is responsible for physiological production of prostacyclin in the cardiovascular system.. Proc Natl Acad Sci U S A.

[7] Liu B, Luo W, Zhang Y, Li H, Zhu N, Huang D, Zhou Y (2012). Involvement of cyclo-oxygenase-1-mediated prostacyclin synthesis in the vasoconstrictor activity evoked by ACh in mouse arteries.. Exp Physiol.

[8] Cheng Y, Wang M, Yu Y, Lawson J, Funk CD, Fitzgerald GA (2006). Cyclooxygenases, microsomal prostaglandin E synthase-1, and cardiovascular function.. J Clin Invest.

[9] Yu Y, Ricciotti E, Scalia R, Tang SY, Grant G, Yu Z, Landesberg G, Crichton I, Wu W, Puré E (2012). Vascular COX-2 modulates blood pressure and thrombosis in mice.. Sci Transl Med.

[10] Mitchell JA, Knowles RB, Kirkby NS, Reed DM, Edin ML, White WE, Chan MV, Longhurst H, Yaqoob MM, Milne GL (2018). Kidney transplantation in a patient lacking cytosolic phospholipase A2 proves renal origins of urinary PGI-M and TX-M.. Circ Res.

[11] Barbieri SS, Amadio P, Gianellini S, Tarantino E, Zacchi E, Veglia F, Howe LR, Weksler BB, Mussoni L, Tremoli E (2012). Cyclooxygenase-2-derived prostacyclin regulates arterial thrombus formation by suppressing tissue factor in a sirtuin-1-dependent-manner.. Circulation.

[12] Bresalier RS, Sandler RS, Quan H, Bolognese JA, Oxenius B, Horgan K, Lines C, Riddell R, Morton D, Lanas A, Adenomatous Polyp Prevention on Vioxx (APPROVe) Trial Investigators (2005). Cardiovascular events associated with rofecoxib in a colorectal adenoma chemoprevention trial.. N Engl J Med.

[13] Armstrong PC, Kirkby NS, Zain ZN, Emerson M, Mitchell JA, Warner TD (2011). Thrombosis is reduced by inhibition of COX-1, but unaffected by inhibition of COX-2, in an acute model of platelet activation in the mouse.. PLoS One.

[14] Doutremepuich C, Aguejouf O, Desplat V, Eizayaga FX (2010). Paradoxical thrombotic effects of aspirin: experimental study on 1000 animals.. Cardiovasc Hematol Disord Drug Targets.

[15] Moncada S, Gryglewski R, Bunting S, Vane JR (1976). An enzyme isolated from arteries transforms prostaglandin endoperoxides to an unstable substance that inhibits platelet aggregation.. Nature.

[16] Zhou J, Wu Y, Chen F, Wang L, Rauova L, Hayes VM, Poncz M, Li H, Liu T, Liu J (2017). The disulfide isomerase ERp72 supports arterial thrombosis in mice.. Blood.

[17] Marcus AJ, Weksler BB, Jaffe EA, Broekman MJ (1980). Synthesis of prostacyclin from platelet-derived endoperoxides by cultured human endothelial cells.. J Clin Invest.

[18] Mitchell JA, Elghazouli Y, Armstrong PC, Vadgama A, Herschman HR, Kirkby NS (2018). Renal or vascular deletion of COX-2 increases thrombotic tone: relevance to cardiovascular side effects of non-steroidal anti-inflammatory drugs.. Circulation.

[19] Li S, Liu B, Luo W, Zhang Y, Li H, Huang D, Zhou Y (2016). Role of cyclooxygenase-1 and -2 in endothelium-dependent contraction of atherosclerotic mouse abdominal aortas.. Clin Exp Pharmacol Physiol.

[20] Kirkby NS, Lundberg MH, Wright WR, Warner TD, Paul-Clark MJ, Mitchell JA (2014). COX-2 protects against atherosclerosis independently of local vascular prostacyclin: identification of COX-2 associated pathways implicate Rgl1 and lymphocyte networks.. PLoS One.

[21] Kirkby NS, Chan MV, Lundberg MH, Massey KA, Edmands WM, MacKenzie LS, Holmes E, Nicolaou A, Warner TD, Mitchell JA (2013). Aspirin-triggered 15-epi-lipoxin A4 predicts cyclooxygenase-2 in the lungs of LPS-treated mice but not in the circulation: implications for a clinical test.. FASEB J.

