# Supplementary figures and images for: Cell-Specific Gene Deletion Reveals the Antithrombotic Function of COX1 and Explains the Vascular COX1/Prostacyclin Paradox
Source: Circ Res. 2019 Sep 12;125(9):847–54. doi: 10.1161/CIRCRESAHA.119.314927 (PMC6791564; doi:10.1161/CIRCRESAHA.119.314927)

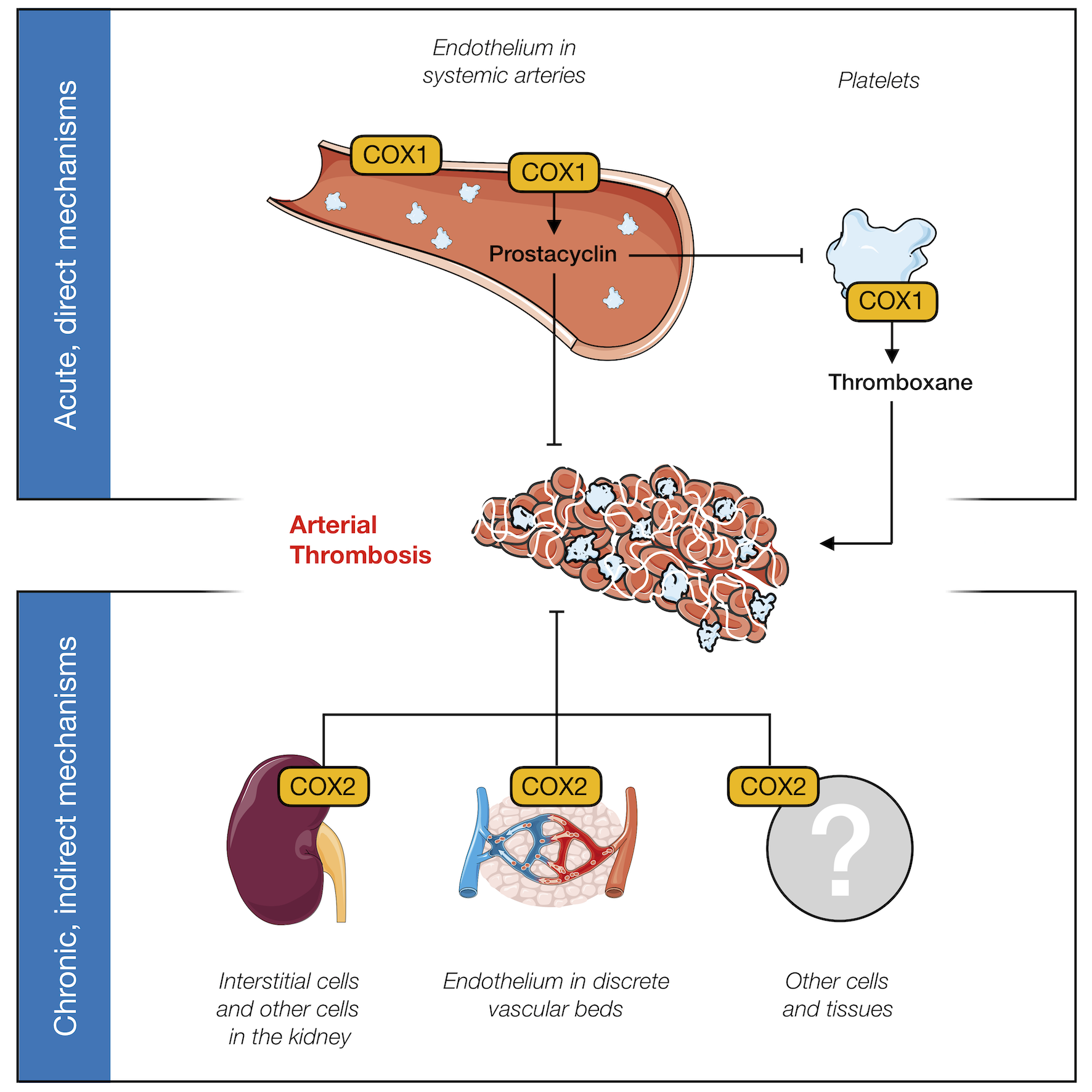

Supplement: Supplementary file 1 [file res-125-847-s001.tif]
