# Supplementary material for: Cell-Specific Gene Deletion Reveals the Antithrombotic Function of COX1 and Explains the Vascular COX1/Prostacyclin Paradox
Source: Circ Res. 2019 Sep 12;125(9):847–54. doi: 10.1161/CIRCRESAHA.119.314927 (PMC6791564; doi:10.1161/CIRCRESAHA.119.314927)
Supplement: Supplementary file 2 [file res-125-847-s002.pdf]

Supplemental Material

Methods

Animals

All experiments were performed on 8-12 week old male and female mice with no specific inclusion/exclusion criteria. *Ptgs1*^{flox} mice were generated by inserting loxP sequences to flank exons 3 and 5 of the *Ptgs1* gene in ES cells, which were injected into blastocysts to create chimeric mice with germline transmission. These animals have been deposited at Jackson Laboratories (USA) as strain no. 030884. *Ptgs1*^{flox} mice were crossed with *VE-cadherin Cre*^{ERT2} mice (generated and provided by Prof Ralf Adams, Max Planck Institute, Germany)[1] to generate EC COX1 KOs (*Ptgs1*$^{flox/flox}$;VE-cadherin-Cre^{ERT2}), *Tie2 Cre*[2] mice to generate EC/PT COX1 KOs (*Ptgs1*$^{flox/flox}$*;Tie2-Cre*) or *Sm22a Cre*[3] mice to generate SMC COX1 KOs (*Ptgs1*$^{flox/flox}$*;Sm22a-Cre). Ptgs2*^{flox} mice[4] (JAX stain no. 030785) were generated as previously described and were crossed with *Tie2 Cre*[2] mice to generate EC COX2 KOs (*Ptgs1*$^{flox/flox}$*;Tie2-Cre*). Although less selective than the *VE-cadherin Cre*^{ERT2} driver, this Cre was chosen to generate EC COX2 KO that were consistent with previous literature[5] and because COX-2 is not expressed in platelets, the activation of Cre in megakaryocytes is inconsequential. Each strain was compared to its own *Cre*-negative littermate control animals and was maintained on a mixed C57Bl/6 and 129S4/SvJae background. As the *Cre*^{ERT2} construct in EC COX1 KO mice requires activation by tamoxifen, at 5-6 weeks of age, these mice and their littermate controls were treated with tamoxifen for 5 days (50mg/kg od, ip; Sigma, UK) and allowed to recover for at least 2 weeks before further use. Additional experiments were performed on germline global COX1 knockout mice[6] (Global KO; *Ptgs*1$^{-/-}$; C57Bl/6 background) which were compared to wild-type controls (Charles River, UK). All mouse strains were viable and showed no gross phenotype or deviation from expected Mendelian distribution of genotypes. Animals were housed with free access to food (RM1; Special Diet Services, UK) and water under a 12h day/night cycle. Procedures were carried out in accordance with the Animals (Scientific Procedures) Act (1986) Amendment Regulations (2012) after review by the Imperial College Animal Welfare and Ethical Review Board and under the authority of UK Home Office license 70/8422. Animals were randomized through allocation of sequential number at weaning (prior to genotyping) and experiments performed in this order. For ex vivo/in vitro tissue experiments, mice were humanely killed by CO_2 narcosis and the vasculature flushed with PBS via the left heart.

COX immunoreactivity

Thoracic aorta was carefully removed, cleaned of peri-adventitial material and divided into 2mm rings. In some cases, aortic rings were cultured in DMEM (Sigma, UK) supplemented with 10% FCS (Labtech, UK) and LPS (1µg/mL; from *E. coli* serotype O111:B4; Sigma, UK) for 24 hrs in a humidified incubator to induce COX2. Fresh or cultured aortic rings were fixed (5% formalin; Sigma, UK), blocked (20% normal goat serum; Abcam, UK) and permeabilised (0.1% Triton X-100; Sigma, UK) then incubated with primary antibodies against mouse COX1 (rabbit anti-mouse polyclonal IgG; Cayman Chemical, USA; catalogue no. 160109; 1:50) or COX2 (rabbit anti-mouse polyclonal IgG; Cayman Chemical, USA; catalogue no. 160126; 1:50) overnight. In each staining experiment, negative controls were included as (1) pre-incubation of the primary antibody with a specific blocking peptide (Cayman Chemical, USA) and (2) omission of the primary antibody.

Primary antibody binding was detected using an Alexa568-conjugated secondary antibody (goat anti-rabbit polyclonal IgG; Life Technologies, UK; catalogue no. A-11036; 1:200) and tissues counterstained using Alexa488-conjugated anti-CD31 (rat anti-mouse monoclonal IgG2a,k; clone MEC13.3; Biolegend, USA; catalogue no. 102514; 1:100) and DAPI (25µg/mL; Life Technologies, UK). Stained aortic rings were cut open and mounted en face between a slide and coverslip using hard-set media (Vector Laboratories, UK). The luminal surface was imaged using a Lecia SP5 inverted confocal microscope and a 63X oil immersion objective. The endothelial and smooth muscle layers were located based on CD31 immunoreactivity and nuclear morphology. Individual images were collected from both layers and in some cases, Z-stacks through the full thickness of the aortic wall were collected. Images were quantified as fluorescent intensity using ImageJ software (NIH, USA) and background corrected by subtracting the signal present when primary antibody was omitted. For any experiment, all image acquisition settings were fixed and any post-processing was applied equally to all images within the dataset. Staining intensity was normalised to 'positive control' conditions stained and imaged on the same day defined as basal expression in a control vessel for COX-1, or induced expression in a control vessel for COX-2. In some cases, Z-stacks were rendered in 3D using Vaa3D software[7-9].

Vessel prostacyclin generation

Thoracic aorta or carotid arteries were carefully removed, cleaned of peri-adventitial material and divided into 2mm rings. In some cases these were denuded of endothelial cells by rubbing the luminal surface with fine forceps. These were incubated at 37°C in DMEM (Sigma, UK) either (1) under static conditions and in the presence of A23187 Ca^{2+} ionophore (30µmol/L; Sigma, UK) or (2) under conditions of physical activation (1300RPM shaking, BioShakeIQ; Q Instruments, Germany) without chemical stimulation. After 30 mins, the supernatant was removed and prostacyclin's spontaneous breakdown product, 6-keto-$PGF_{1\alpha}$, measured by immunoassay (Enzo Lifesciences, USA or Cayman Chemical, USA).

Plasma prostacyclin and whole blood thromboxane generation

Whole blood was collected from the inferior vena cava into heparin (10U/mL final; Leo Laboratories, UK). Thromboxane generation was determined by stimulating whole blood with A23187 (30µmol/L) for 30 mins at 37°C, then separating conditioned plasma by centrifugation (8000g, 2 mins) and measuring the stable thromboxane A_2 breakdown product, thromboxane B_2, by immunoassay (Cayman Chemical, USA). Prostacyclin was measured as 6-keto-$PGF_{1\alpha}$ by immunoassay (Cayman Chemical, USA) in plasma separated by centrifugation (8000g, 2 mins) from unstimulated blood.

Aortic endothelial and smooth muscle cell isolation

Thoracic and abdominal aorta were rigorously cleaned of adherent material, divided into 1mm rings and digested at 37°C for 1 hr in an enzyme cocktail containing collagenase type I (5mg/mL; Sigma, UK), elastase (100µg/mL; Sigma, UK) and DNase I (125U/mL; Sigma, UK), $CaCl_2$ (1µmol/L). To prevent artefactual changes in COX expression, the protein synthesis inhibitor cycloheximide (3µmol/L; Sigma, UK) was included in this and all subsequent steps. The resulting suspension was passed through a 40µm cell strainer, treated with Red Cell Lysis buffer (Sigma, UK) and blocked with FcR blocking reagent (Miltenyi Biotec, UK). Cells

were then stained using anti-CD31-PerCP/Cy5.5 (rat anti-mouse monoclonal IgG2a,k; clone MEC13.3; catalogue no. 102522; Biolegend, USA; 1:20), anti-CD45-PE/Vio770 (recombinant human anti-mouse monoclonal IgG1; clone REA737; Miltenyi Biotec, UK; catalogue no. 130-110-661; 1:50) and anti-CD41-APC/Cy7 (rat anti-mouse monoclonical IgG1,k; clone MWReg30; Biolegend, UK; catalogue no. 133928; 1:20), then re-suspended in DAPI (5µg/ml). The stained cell suspension was sorted using a FACSAria III instrument (BD Biosciences, Germany), gated to exclude sub-cellular debris (FSC vs SSC), cell doublets (FSC-A vs. FSC-H), non-viable cells ($DAPI^+$), leucocytes ($CD45^+$), platelets and platelet-bound cells ($CD41^+$). The remaining population was collected using a 2-way purity sort and 100µm nozzle with $CD31^+$ cells defined as endothelial and remaining $CD31^-$, $CD41^-$, $CD45^-$ cells considered to be smooth muscle cells. Sorted cells were resuspended in DMEM containing arachidonic acid (30µmol/L; Sigma, UK), incubated for 20 mins at 37°C, then the supernatant collected to measure 6-keto-$PGF_{1\alpha}$ by immunoassay (Cayman Chemical, USA).

Human platelet bioassay for prostacyclin activity

Blood was collected from human healthy volunteers by forearm venepuncture into trisodium citrate (0.32% final; Sigma, UK) after giving informed consent. This was approved by the West London & GTAC Research Ethics Committee as study 15/LO/223. Platelet-rich plasma (PRP) was separated by centrifugation (170g, 15 mins) and platelet-poor plasma (PPP) by further centrifugation of PRP (8000g, 2 mins). PRP was pre-treated with aspirin (30µmol/L; Sigma, UK; 30 mins) and DEA/NONOate (10µmol/L; Sigma, UK; 2 mins) to block platelet COX activity and sensitize platelets to prostacyclin. In some cases (Online Figure V), aspirin was replaced with the thromboxane receptor antagonist, terutroban (10µmol/L; Tocris Bioscience, UK), then the effect of additionally adding aspirin determined. In all cases, PRP was then added to 96 well microtitre plates containing cleaned mouse aortic rings and incubated at 37°C for 1 min. Platelet aggregation was stimulated by addition of A23187 (30µmol/L) and vigorous mixing (1200RPM) for 5 mins essentially as we have previously described[10]. Aortic rings were then quickly removed and the absorbance of each well at 620nm measured using a spectrophotometric plate reader (Tecan, Germany). Platelet aggregation was calculated according to the principle of light transmission aggregometry, using the absorbance of unstimulated PRP and PPP as reference to 0% and 100% aggregation, respectively.

In vivo thrombosis model

Mice were anaesthetised with isoflurane (Abbot Laboratories, USA) and maintained at 37°C using a homeothermic heated stage and secured in a supine position. An incision was made over the neck midline and the left carotid artery isolated and separated from the attached nerve, vein and surrounding tissue by careful blunt dissection. Basal carotid artery blood flow was measured over 5 mins by securing a Doppler flow probe (Transonic, USA) around the artery. The probe was then removed while a filter paper saturated with 4-6% ferric chloride solution (Sigma, UK) was placed to be in contact with the dorsal, adventitial surface of the carotid artery for 3 mins. After this, the site was rinsed in saline and flow probe re-attached. Blood flow was monitored over 20 mins and the time taken for thrombotic occlusion (defined as flow <0.2ml/min) recorded. Where no occlusion was observed over this period, occlusion time was recorded as 20 mins. In some studies, 20 mins before performing carotid artery injury, mice were administered the selective IP receptor antagonist Ro1138452 (10mg/kg; iv; R&D Systems, UK) or its vehicle (5% DMSO, Sigma, UK). In

other studies, mice were treated with parecoxib (Pfizer, USA), either (i) acutely, by intravenous injection (5mg/kg), 20 mins prior to carotid artery injury or (ii) continuously by addition to drinking water (25mg/kg/day) for 5 days prior to carotid artery injury.

COX-2 inhibition assay

An ex vivo bioassay[11] was used to assess the levels of COX-2 inhibitory activity of plasma from mice treated with parecoxib by different routes and for different durations. Plasma from parecoxib and vehicle-treated mice was applied to J774.2 cells (Sigma, UK; authenticated by the European Collection of Authenticated Cultures; experiments performed at passage 3 from purchased stock) pre-treated with LPS to induce COX-2 (1µg/mL; from *E. coli* serotype O111:B4; Sigma, UK; 24 hrs). Cells were activated by addition of A23187 Ca^{2+} ionophore (30µmol/L; Sigma, UK) and after 30 mins, conditioned plasma collected for measurement by immunoassay (Cisbio, France) of the levels of COX-2-derived PGE_2 released by the cells.

Statistics and data analysis

Data were analysed using Prism 8 software (GraphPad, USA) and are presented at mean ± standard error for n biological replicates. Where multiple measurements of the same endpoint were made from the same animal these were averaged and considered n=1. In most cases, only a single experimental endpoint was studied from an individual animal. Wherever reasonably possible, samples/data were collected and analysed by an investigator blinding to the animal genotype/treatment and tracked only using animal numbers allocated prior to genotyping. Study sample size was based on Cohen's D effect size estimate for the endpoints under study derived from previous experience with these models. Statistical tests and multiple comparisons corrections within each data set are detailed in the respective figure legends. No correction for multiple comparisons across different datasets/endpoints has been made, which may be considered a limitation of the statistical approach. Differences were considered significant where $p<0.05$ (two-sided). Distribution of data was assessed using Shapiro-Wilk test and where n was sufficient to allow, used to inform the choice of parametric versus non-parametric statistical test. The data that support the findings of this study are available from the corresponding author upon reasonable request.

1. Sorensen I, Adams RH and Gossler A. DLL1-mediated Notch activation regulates endothelial identity in mouse fetal arteries. *Blood*. 2009;113:5680-8.
2. Kisanuki YY, Hammer RE, Miyazaki J, Williams SC, Richardson JA and Yanagisawa M. Tie2-Cre transgenic mice: a new model for endothelial cell-lineage analysis in vivo. *Dev Biol*. 2001;230:230-42.
3. Holtwick R, Gotthardt M, Skryabin B, Steinmetz M, Potthast R, Zetsche B, Hammer RE, Herz J and Kuhn M. Smooth muscle-selective deletion of guanylyl cyclase-A prevents the acute but not chronic effects of ANP on blood pressure. *Proc Natl Acad Sci U S A*. 2002;99:7142-7.
4. Ishikawa TO and Herschman HR. Conditional knockout mouse for tissue-specific disruption of the cyclooxygenase-2 (Cox-2) gene. *Genesis*. 2006;44:143-9.
5. Yu Y, Ricciotti E, Scalia R, Tang SY, Grant G, Yu Z, Landesberg G, Crichton I, Wu W, Pure E, Funk CD and FitzGerald GA. Vascular COX-2 modulates blood pressure and thrombosis in mice. *Sci Transl Med*. 2012;4:132ra54.

6. Langenbach R, Morham SG, Tiano HF, Loftin CD, Ghanayem BI, Chulada PC, Mahler JF, Lee CA, Goulding EH, Kluckman KD, Kim HS and Smithies O. Prostaglandin synthase 1 gene disruption in mice reduces arachidonic acid-induced inflammation and indomethacin-induced gastric ulceration. *Cell*. 1995;83:483-92.
7. Peng H, Ruan Z, Long F, Simpson JH and Myers EW. V3D enables real-time 3D visualization and quantitative analysis of large-scale biological image data sets. *Nat Biotechnol*. 2010;28:348-53.
8. Peng H, Bria A, Zhou Z, Iannello G and Long F. Extensible visualization and analysis for multidimensional images using Vaa3D. *Nat Protoc*. 2014;9:193-208.
9. Peng H, Tang J, Xiao H, Bria A, Zhou J, Butler V, Zhou Z, Gonzalez-Bellido PT, Oh SW, Chen J, Mitra A, Tsien RW, Zeng H, Ascoli GA, Iannello G, Hawrylycz M, Myers E and Long F. Virtual finger boosts three-dimensional imaging and microsurgery as well as terabyte volume image visualization and analysis. *Nat Commun*. 2014;5:4342.
10. Armstrong PC, Dhanji AR, Truss NJ, Zain ZN, Tucker AT, Mitchell JA and Warner TD. Utility of 96-well plate aggregometry and measurement of thrombi adhesion to determine aspirin and clopidogrel effectiveness. *Thromb Haemost*. 2009;102:772-8.
11. Kirkby NS, Lundberg MH, Harrington LS, Leadbeater PD, Milne GL, Potter CM, Al-Yamani M, Adeyemi O, Warner TD and Mitchell JA. Cyclooxygenase-1, not cyclooxygenase-2, is responsible for physiological production of prostacyclin in the cardiovascular system. *Proc Natl Acad Sci U S A*. 2012;109:17597-602.

Figures & Tables

**Strain**	**Basal COX1**	**Basal COX2**	**Induced COX2**
	fold vs. respective control strain	*fold vs. induced COX2 level in respective control strain*	
Flox COX1 Ctrl	1.00 ± 0.14 *n=10*	0.07 ± 0.04 *n=6*	1.00 ± 0.21 *n=6*
EC COX1 KO	0.21 ± 0.06 $^{\#}$ *n=5; p<0.001*	0.02 ± 0.01 *n=3; p=0.780*	0.93 ± 0.42 *n=3; p=0.978*
EC/PT COX1 KO	0.15 ± 0.06 $^{\#}$ *n=7; p<0.001*	0.15 ± 0.08 *n=4; p=0.440*	0.70 ± 0.23 *n=4; p=0.660*
Wild-type Ctrl	1.00 ± 0.30 *n=3*	0.24 ± 0.23 *n=3*	1.0 ± 0.43 *n=4*
Global COX1 KO	0.05 ± 0.03 * *n=3; p=0.034*	0.13 ± 0.11 *n=3; p=0.675*	1.20 ± 0.11 *n=4; p=0.599*
Flox COX2 Ctrl	1.00 ± 0.08 *n=7*	0.14 ± 0.06 *n=8*	1.00 ± 0.15 *n=6*
EC COX2 KO	1.30 ± 0.26 *n=4; p=0.192*	0.20 ±0.06 *n=4; p=0.609*	0.19 ± 0.10 * *n=4; p=0.003*
Blocking peptide	0.14 ± 0.05 *n=6*	0.07 ± 0.04 *n=6*	

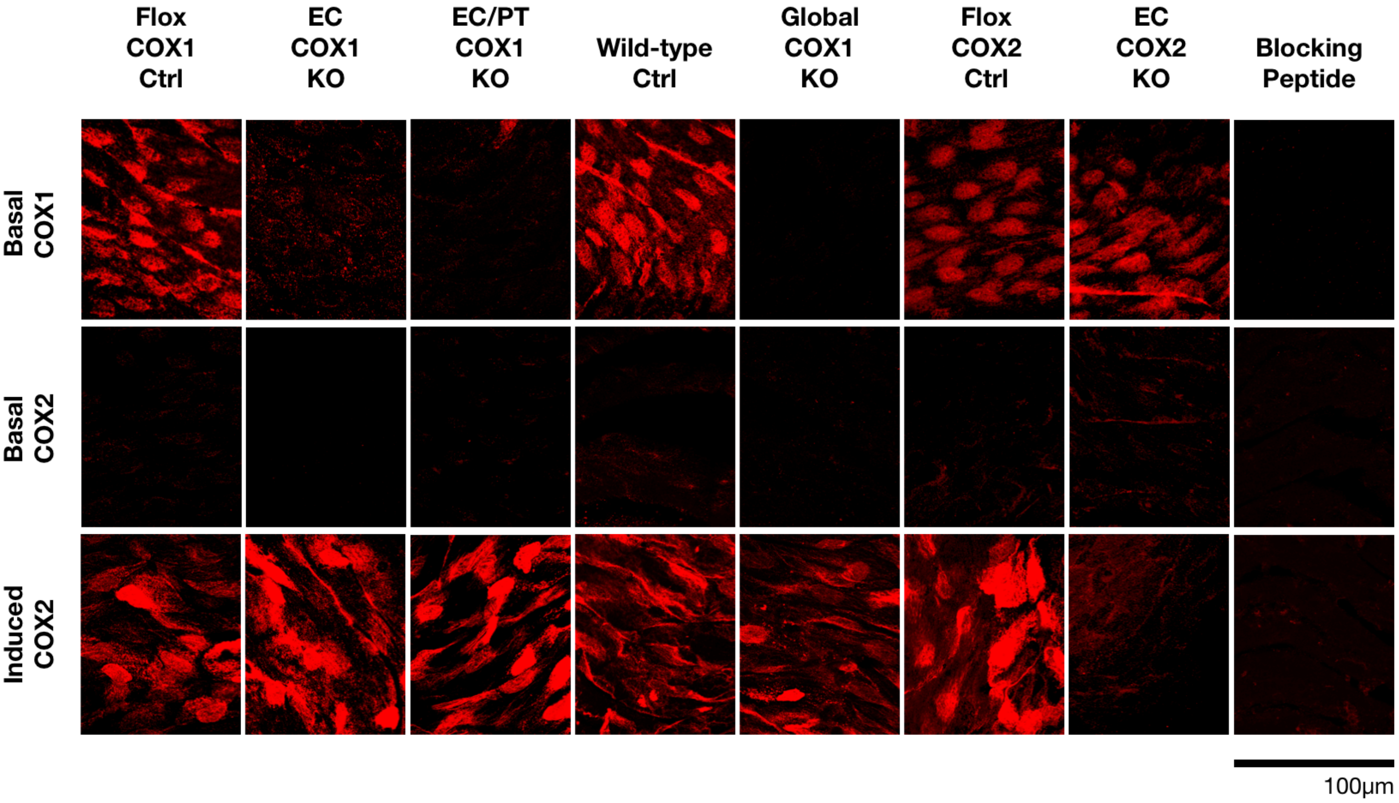

Online Figure I. Quantification of COX1 and COX2 immunoreactivity in the aortic endothelium of conditional COX1 and COX2 knockout (KO) strains. Staining quantified as fluorescence intensity and normalised to respective control strains stained/imaged in the same experiment within each antibody condition. Representative images show COX1 / COX2 immunoreactivity and are scaled within each antibody for comparison between strains/conditions. *, $p<0.05$ by unpaired t-test, $^{\#}$, $p<0.05$ by Kruskal-Wallis ANOVA with Sidak's post-test versus respective control strain.

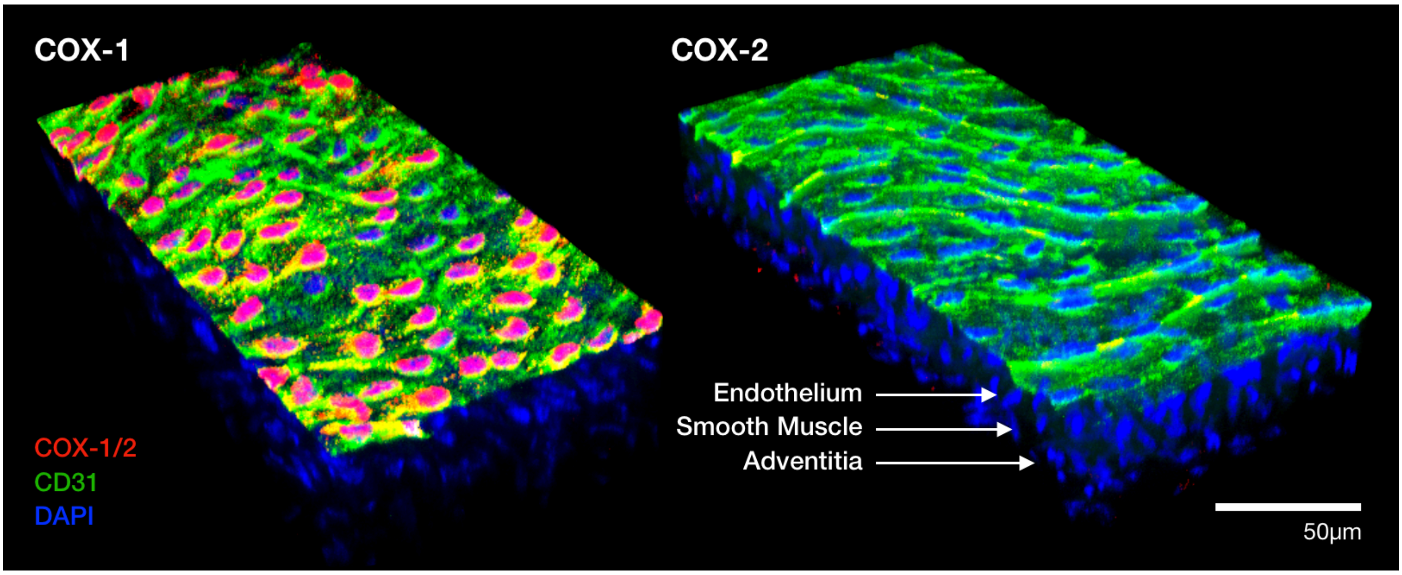

Online Figure II. Pattern of COX1 and COX2 immunoreactivity through the full thickness of the aortic wall. Representative rendered images from confocal Z-stacks of Flox COX1 Ctrl aorta.

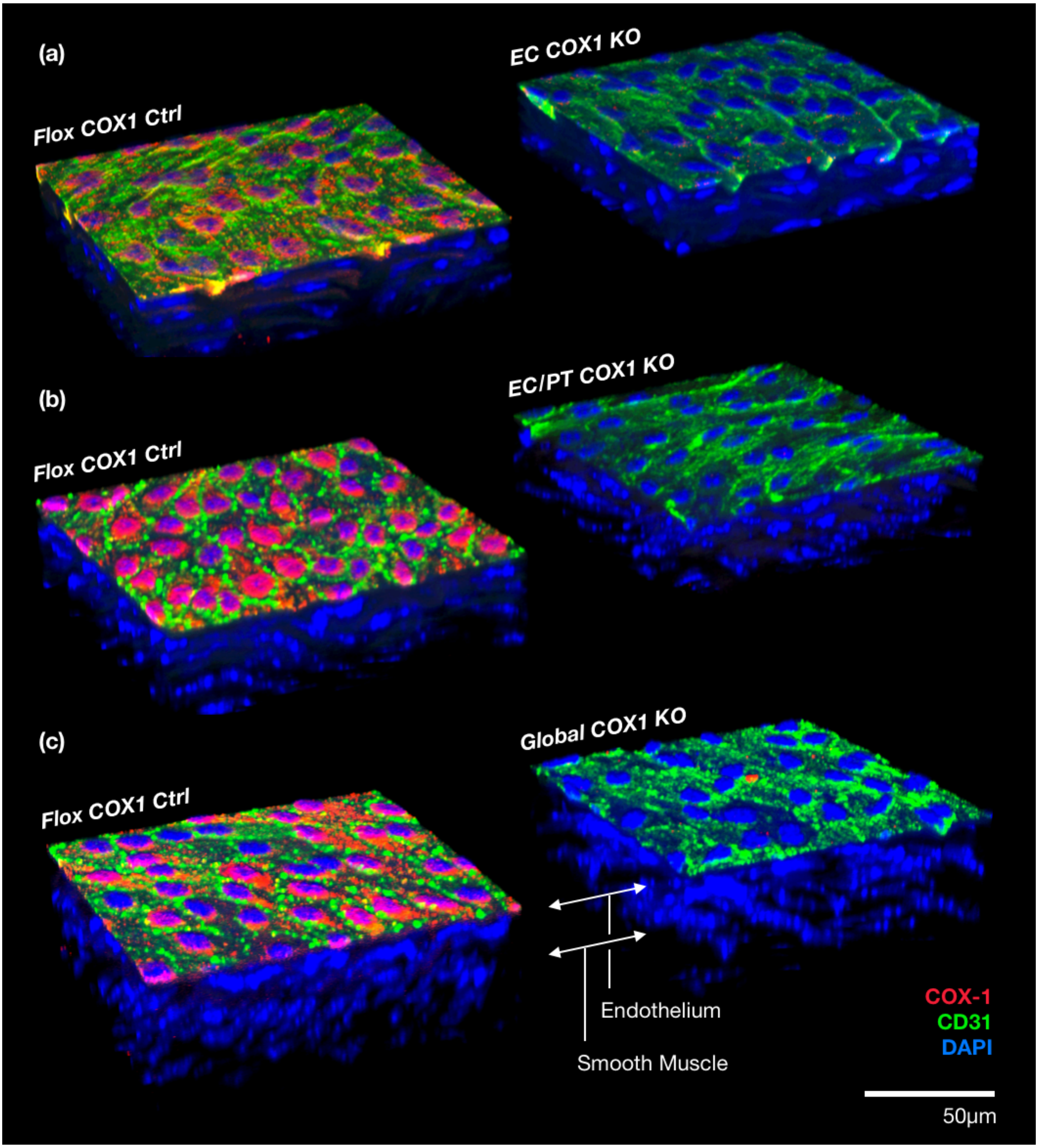

Online Figure III. Pattern of COX1 immunoreactivity through the full thickness of the aortic wall in studied COX1 knockout (KO) strains and controls. Representative rendered images from confocal Z-stacks of aorta from (a) Flox COX1 Ctrl and EC COX1 KO, (b) Flox COX1 Ctrl and EC/PT COX1 KO and (c) Flox COX1 Ctrl and Global COX1 KO mice.

Strain	Blood [TXB_2] ng/mL	Aorta [$6kPGF_{1\alpha}$] A23187 ng/mL	Aorta [$6kPGF_{1\alpha}$] Physical ng/mL	Aorta anti-platelet activity %	Plasma [$6kPGF_{1\alpha}$] pg/mL
Flox COX1 Ctrl	208 ± 15 *n=5*	6.3 ± 1.6 *n=12*	7.4 ± 0.6 *n=6*	100 ± 10 *n=4*	324 ± 29 *n=5*
EC COX1 KO	196 ± 7 *n=5; p=0.483*	1.6 ± 0.4 # *n=9; p=0.006*	3.4 ± 0.7 * *n=6; p=0.002*	20 ± 7 * *n=5; p<0.001*	190 ± 16 * *n=8; p=0.001*
Flox COX1 Ctrl	193 ± 6 *n=4*	3.7 ± 0.7 *n=4*	7.3 ± 1.9 *n=4*	100 ± 6 *n=7*	199 ± 27 *n=4*
EC/PT COX1 KO	22 ± 12 * *n=4; p<0.001*	0.8 ± 0.0 * *n=4; p=0.019*	2.0 ± 0.2 * *n=4; p=0.032*	37 ± 6 * *n=4; p=0.001*	96 ± 18 * *n=4; p=0.019*
Wild-type Ctrl	185 ± 25 *n=5*	3.8 ± 0.8 *n=6*	-	100 ± 24 *n=5*	307 ± 43 *n=6*
Global COX1 KO	4 ± 0 * *n=5; p<0.001*	0.1 ± 0.0 * *n=6; p=0.001*	-	3 ± 2 # *n=5; p=0.008*	43 ± 3 # *n=3; p=0.024*
Flox COX2 Ctrl	240 ± 28 *n=6*	9.3 ± 1.3 *n=12*	14.8 ± 2.1 *n=10*	100 ± 22 *n=3*	363 ± 69 *n=10*
EC COX2 KO	228 ± 29 *n=7; p=0.604*	10.3 ± 0.6 *n=9; ; p=0.239*	12.1 ± 1.3 *n=7; p=0.343*	97 ± 12 *n=5; p=0.892*	300 ± 64 *n=6; p=0.549*

Online Table I. Prostanoid production and aortic anti-platelet activity in COX1 and COX2 knockout (KO) strains. *, $p<0.05$ by unpaired t-test, #, $p<0.05$ by Mann-Whitney U-test versus respective control strain.

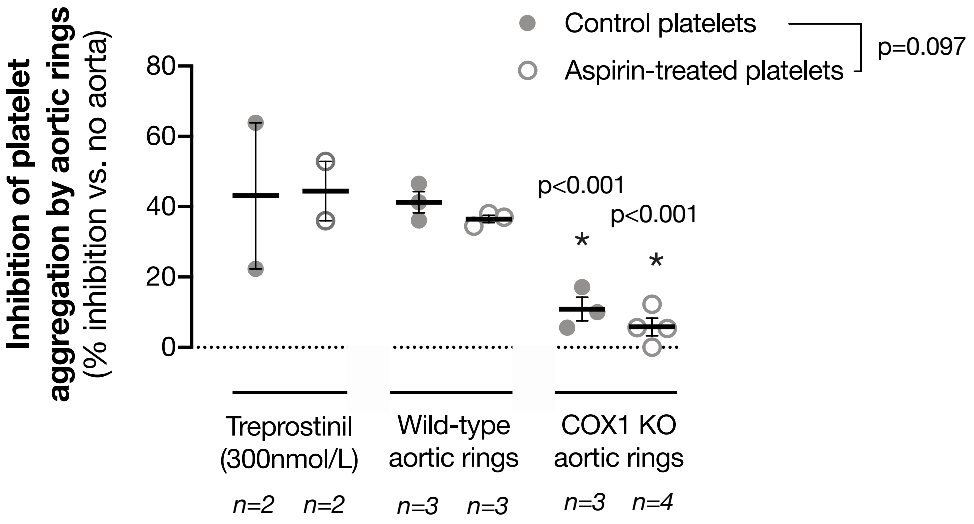

Online Figure IV. Contribution of platelet COX1-derived intermediates to the inhibition of aggregation produced by aortic rings ex vivo. Wild-type aortic rings (n=3) produced inhibition of platelet aggregation which was lost when aortic rings from Global COX1 KO mice (n=3-4) were used. The responses were not altered when platelets were pre-treated with aspirin (30µmol/L) to prevent platelet PGH_2 generation. This suggests that in this assay, platelet-derived PGH_2 cannot be donated to endothelial cells to produce anti-platelet prostacyclin, even when vascular COX1 is absent. The platelet inhibitory effect prostacyclin analogue, treprostinil (300nmol/L, n=2) is shown for reference. *, p<0.05 versus wild-type aorta by two-way ANOVA with Sidak's post-test.

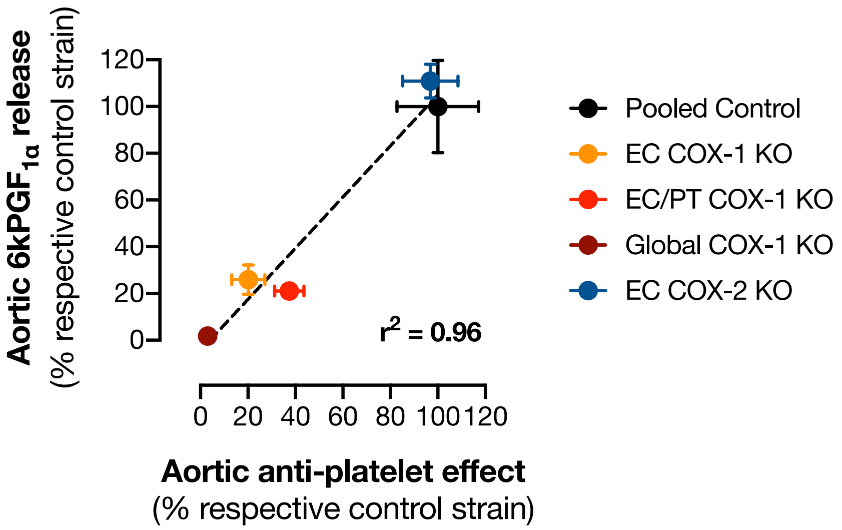

Online Figure V. Correlation of prostacyclin production and anti-platelet activity of aortic rings from studied COX1 and COX2 knockout (KO) strains. Data are mean ± SEM for n=4-9 animals, plotted from raw values in Online Figure IV.

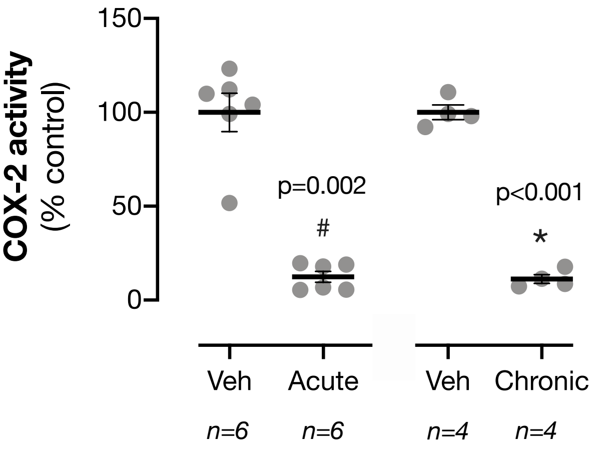

Online Figure VI. COX2 inhibition by parecoxib by different routes of administration. Mice were dosed with parecoxib acutely (‘Acute’; 5mg/kg; iv; 20 mins) or chronically (‘Chronic’; 25mg/kg/day; po; 5 days) and COX2 inhibitory activity of plasma bioassayed on COX2 expressing cells ex vivo. *, $p<0.05$ by unpaired t-test, $^{\#}$, $p<0.05$ by Mann-Whitney U-test versus vehicle.
